# Recovery of Selenium-Enriched Polysaccharides from *Cardamine violifolia* Residues: Comparison on Structure and Antioxidant Activity by Different Extraction Methods

**DOI:** 10.3390/antiox13101251

**Published:** 2024-10-17

**Authors:** Yong Liang, Jiali Yu, Lulu Wu, Xin Cong, Haiyuan Liu, Xu Chen, Shuyi Li, Zhenzhou Zhu

**Affiliations:** 1 School of Modern Industry for Selenium Science and Engineering, National R&D Center for Se-Rich Agricultural Products Processing Technology, Wuhan Polytechnic University, Wuhan 430023, China; liangyong358156@163.com (Y.L.); yyyu8989@163.com (J.Y.); lulu672024@163.com (L.W.); congxinwhpu@whpu.edu.cn (X.C.); 7240111024@stu.jiangnan.edu.cn (H.L.); chxu@whpu.edu.cn (X.C.); 2 Hubei Engineering Research Center for Deep Processing of Green Se-Rich Agricultural Products, Wuhan 430023, China

**Keywords:** SePS, ultrasound-assisted extraction, structural characterization, antioxidant activity

## Abstract

The residues from selenium-enriched *Cardamine violifolia* after the extraction of protein were still rich in polysaccharides. Thus, the recovery of selenium polysaccharides (SePSs) was compared using hot water extraction and ultrasonic-assisted extraction techniques. The yield, extraction rate, purity, specific energy consumption, and content of total and organic selenium from different SePS extracts were determined. The results indicated that at conditions of 250 W (ultrasonic power), 30 °C, and a liquid-to-material ratio of 30:1 extracted for 60 min, the yield of SePSs was 3.97 ± 0.07%, the extraction rate was 22.76 ± 0.40%, and the purity was 65.56 ± 0.35%, while the total and organic selenium content was 749.16 ± 6.91 mg/kg and 628.37 ± 5.93 mg/kg, respectively. Compared to traditional hot water extraction, ultrasonic-assisted extraction significantly improves efficiency, reduces energy use, and boosts both total and organic selenium content in the extract. Measurements of particle size, molecular weight, and monosaccharide composition, along with infrared and ultraviolet spectroscopy, revealed that ultrasonic-assisted extraction breaks down long-chain structures, decreases particle size, and changes monosaccharide composition in SePSs, leading to lower molecular weight and reduced dispersity. The unique structure of SePSs, which integrates selenium with polysaccharide groups, results in markedly improved antioxidant activity and reducing power, even at low concentrations, due to the synergistic effects of selenium and polysaccharides. This study establishes a basis for using SePSs in functional foods.

## 1. Introduction

Selenium is a vital trace element essential for human health [1]. Studies indicate that the organic selenium compounds including selenoprotein and selenomethionine, selenocysteine, and methylselenocysteine can be more readily absorbed and utilized by the human body compared to inorganic selenium forms, such as sodium selenite and sodium selenate, which promotes the application of organic selenium and gains increased attention [1,2]. Selenium-enriched *Cardamine violifolia* is a hyperaccumulator plant native to the Enshi region of China. This plant has a high capacity to accumulate and tolerate selenium which has been consumed in Enshi for a long time. Its tender and nutritious stems and leaves effectively convert inorganic selenium into organic forms, of which the total selenium content can reach 2000–9000 mg/kg [3]. Recently, scientists have successfully separated selenoproteins from selenium-enriched *Cardamine violifolia* and found promising functions related to these extracts. For example, Tian et al. have demonstrated that the selenium-enriched *Cardamine violifolia* peptides (CSPs) significantly alleviated obesity and related metabolic disorders induced by a high-fat diet in mice. Moreover, CSPs could maintain intestinal integrity by regulating gut microbiota and reducing oxidative stress and inflammation [4]. Wang et al. found in the in vitro experiments that selenium-enriched CSPs significantly increased cell survival and proliferation, which also reduced lactate dehydrogenase (LDH) activity, and enhanced tight junctions between cells, improving cell barrier integrity. This indicates that CSPs can promote cell growth, protect cells from damage, and maintain normal cell function [5]. Recently, our team found that the selenoprotein extraction residues from *Cardamine violifolia* still contain high levels of organic selenium compounds, mainly in the form of selenium-containing polysaccharides. SePSs are complexes of selenium and polysaccharides and are an important form of organic selenium, where selenium and polysaccharides are connected by covalent bonds. SePSs exist in various forms, including natural, chemically synthesized, microbially transformed, and plant-transformed SePSs, possessing unique biological activities [6,7]. However, research on SePSs from *Cardamine violifolia* is limited and requires further in-depth study.

At present, numerous methods exist for the extraction of polysaccharides. These include traditional solvent extraction techniques (like water extraction or alkali extraction) [8], microwave-assisted extraction [9], enzymatic-assisted extraction [10], ultrasonic-assisted extraction [11], and two or three methods of synergistic extraction [12]. These methods are extensively utilized in research, yielding notable outcomes. Nonetheless, these methods are associated with several challenges, such as suboptimal extraction efficiency, elevated costs, significant energy demands, rigorous equipment specifications, and intricate procedures. To be mentioned, SePSs require low-temperature extraction to preserve their bioactivity and structural integrity. This compound, rich in selenium, exhibits antioxidative and immunoregulatory functions. Elevated temperatures may compromise its structural integrity and diminish its bioactivity. Lower temperatures contribute to the stability and purity of the compound. Consequently, extracting at low temperatures is essential to maintain the unique properties of SePSs [13]. Thus, research into polysaccharides derived from selenium-enriched *Cardamine violifolia* is still nascent, with numerous challenges yet to be addressed. Even though some studies have reported on its pharmacological activity, more comprehensive research is required to fully uncover its potential and applicative value.

This research focused on extracting SePSs from *Cardamine violifolia*. residue, utilizing both hot water and ultrasonic-assisted methods to determine the most effective extraction conditions. The efficacy of each method was assessed by comparing yield, extraction rate, and purity. Additionally, the study examined how these methods influenced the specific energy consumption and selenium content, both total and organic, in the SePSs. Moreover, the impact of ultrasonic-assisted extraction on the SePS physicochemical attributes and structure was investigated through analyses of particle size, molecular weight, monosaccharide composition, and spectral features. The final phase involved evaluating the antioxidant activities of SePSs with different selenium levels, thereby supplying experimental data for analyzing SePS interactions.

## 2. Materials and Methods

### 2.1. Material, Reagents, and Pretreatment

The residue of *Cardamine violifolia* was sourced from Enshi Se-Run Material Engineering Technology Co., Ltd., Enshi, China. Fresh leaves were selected, thoroughly washed, and air-dried. The dried leaves were ground into a fine powder (100 mesh). Distilled water was added at a 1:20 ratio, and the mixture was stirred at 70 °C for 2 h. The extract was centrifuged at 4000 rpm for 20 min, and the supernatant was concentrated as the selenium protein extract. The solid residue was collected, washed several times, and air-dried for further experiments. Dextrose (180 Da) and dextran molecular weight standards (Mw 2700, 9750, 135,030, and 300,600 Da) were sourced from the China National Institute for Food and Drug Control. Fucose (Fuc), rhamnose (Rha), arabinose (Ara), galactose (Gal), galacturonic acid (Gala), glucose (Glc), xylose (Xyl), mannose (Man), and glucuronic acid (Glca) were acquired from BoRui Saccharide Biotech Co., Ltd., (Yangzhou, China). A 1000 μg/mL selenium standard solution, selenite standard solution, and selenate standard solution were procured from the National Standard Material Network (China). The hydroxyl radical, DPPH radical, and ABTS radical were sourced from Beijing Solarbio Science & Technology Co., Ltd. (Beijing, China). Other reagents used were analytical or chromatographic reagents.

An A580 UV-Vis spectrophotometer was procured from Aoyi Instruments (Shanghai, China) Co., Ltd. An iANTO NPAARMONOWAVE 300 microwave digestion instrument was sourced from Anton Paar (Shanghai, China) Trading Co., Ltd. A G-400 intelligent temperature control electric heater acid removal instrument was acquired from Shanghai Yiyao Instrument Technology Development Co., Ltd. (Shanghai, China). A HyLight liquid phase atomic fluorescence spectrometer was supplied by Beijing HyLight Instrument Co., Ltd. (Beijing, China). An inductively coupled plasma mass spectrometer was obtained from Agilent Technologies, Inc., USA (Santa Clara, CA, USA). A NEXUS670 Fourier transform infrared spectrometer was sourced from Nicolet Company, USA (Green Bay, WI, USA). A Waters 2695 high performance liquid chromatograph was procured from Waters Corporation, USA (Milford, MA, USA). A Malvern Mastersizer 3000 laser particle size analyzer was obtained from Malvern Instruments Ltd., UK (Malvern, UK).

### 2.2. Extraction of SePSs by Hot Water Method

A total of 5.0 g of selenium-enriched *Cardamine violifolia* residue powder was measured out, dissolved in a specific ratio of distilled water, and then placed on a magnetic stirrer for complete dissolution, followed by hot water extraction. In the single-factor experimental design, various liquid-to-material ratios (10:1–40:1, *v*/*w*), extraction temperatures (30–90 °C), and extraction durations (10–80 min) were evaluated for their impact on the yield, extraction rate, and purity of SePSs derived from selenium-enriched *Cardamine violifolia*. The extraction solution underwent centrifugation at 4000× *g* for 20 min, after which the supernatant was collected. The solution was then concentrated using rotary evaporation, followed by the addition of four times the volume of anhydrous ethanol solution. Subsequently, alcohol precipitation was performed at 4 °C for 24 h. The mixture was then centrifuged again (4000× *g*, 20 min) to eliminate the ethanol solution. Distilled water was used to completely dissolve the obtained selenium-enriched *Cardamine violifolia* selenium polysaccharide precipitate. The solution was then concentrated and any residual ethanol solution was removed. Lastly, a freeze dryer was utilized to prepare dried selenium-enriched *Cardamine violifolia* SePSs.

### 2.3. Extraction of SePSs by Ultrasonic-Assisted Method

A total of 5.0 g of selenium-enriched *Cardamine violifolia* residue powder was measured out and dissolved it in a specific ratio of distilled water. The solution was then placed on a magnetic stirrer to ensure complete dissolution, followed by ultrasonic-assisted extraction. In the single-factor experimental design, various liquid-to-material ratios (10:1–40:1, *v*/*w*), extraction powers (200–350 W), and extraction durations (10–80 min) were evaluated for their impact on the yield, extraction rate, and purity of SePSs. Upon completion of the extraction, the solution underwent centrifugation at 4000× *g* for 20 min, after which the supernatant was collected. The solution was then concentrated using rotary evaporation, followed by the addition of four times the volume of anhydrous ethanol solution. The solution then underwent alcohol precipitation at 4 °C for 24 h. The mixture was then centrifuged again (4000× *g*, 20 min) to eliminate the ethanol solution. The obtained polysaccharide precipitate was then completely dissolved in distilled water, concentrated, and any residual ethanol solution was removed. Lastly, a freeze dryer was utilized to prepare dried SePSs.

### 2.4. Evaluation of the Extraction Efficiency of SePSs

The content of SePSs was determined by the phenol–sulfuric acid method, and D-glucose was selected as the standard for comparison. The calculation formulas for the yield [14], extraction rate [15], and extraction purity [16] of SePSs are as follows:

(1) $$\mathrm{Yield}\left(\%\right)=M_{0}/M_{1}\times 100$$

(2) $$\mathrm{Extraction}\;\mathrm{rate}\left(\%\right)=M_{0}/M_{2}\times 100$$

(3) $$\mathrm{Purity}\left(\%\right)=M_{0}/M_{3}\times 100$$

In the formula, M_0_ refers to the mass of SePSs extracted from the raw material. M_1_ refers to the total mass of the raw material. M_2_ refers to the total mass of SePSs in the raw material. M3 refers to the total mass of the extract.

The equation for the specific energy consumption in the hot water extraction is as follows:

(4) $$Q_{1}=\frac{m\times c\times (T_{1}-T_{2})}{10^{3}\times V\times M}$$

The equation for the specific energy consumption in the ultrasonic-assisted extraction is as follows:

(5) $$Q_{1}=\frac{P\times t}{V\times M}$$

In the formula, Q represents specific energy consumption, kJ/mg; m is the mass of the liquid heated in the water bath, kg; c is the specific heat capacity of water, 4.18 kJ/(kg.°C); T_1_ is the heating temperature, °C; T_2_ is the initial temperature, °C; V is the extraction volume, mL; M is the content of SePSs in the extract, mg/mL; P is the power of ultrasound, W; and t is the processing time, s.

### 2.5. Composition Analysis of SePS Extract

The phenol–sulfuric acid method, using glucose as the standard [17], was employed to determine the content of neutral sugars in SePSs. The m-hydroxydiphenyl method, with galacturonic acid as the standard [18], was utilized to ascertain the content of uronic acids. The 3,5-dinitrosalicylic acid method, using glucose as the standard [19], was employed to determine the content of reducing sugars. The BCA method, with bovine serum albumin as the standard [20], was utilized to ascertain the protein content. The Folin–Ciocalteu colorimetric method [21] was employed to determine the content of polyphenols. Atomic fluorescence spectrometry [22] was utilized to ascertain the total selenium content. Inductively coupled plasma mass spectrometry [23] was employed to determine the inorganic selenium content.

### 2.6. Particle Size Distribution

A micrometer-level Malvern laser particle size analyzer was employed to ascertain the particle size and distribution of SePSs. The sample was prepared as a 10 mg/mL solution, possessing a refractive index of 1.565 and an absorption rate of 0.01. The dispersant utilized was water, which had a refractive index of 1.33.

### 2.7. Molecular Weight Determination

A Waters 2695 high-performance liquid chromatograph, equipped with a 2410 differential refractometer and Empower workstation, along with an Ultrahydrogel TMLinear 300 mm × 7.8 mm chromatographic column, was utilized to ascertain the molecular weight of SePSs. The mobile phase consisted of a 0.1 M sodium nitrate aqueous solution, with a flow rate of 0.5 mL/min, a column temperature of 40 °C, and a sample volume of 20 μL (1 mg/mL). The molecular weight of SePSs was computed based on the retention time of dextran molecular weight standards, which had varying molecular weights of glucose (180 Da) and (Mw of 2700, 9750, 135,030, and 300,600 Da).

### 2.8. Monosaccharide Composition Determination

The assessment of monosaccharide composition primarily relied on a slightly modified version of Xu’s method [24]. Initially, the selenium polysaccharide samples underwent hydrolysis. The specific steps included measuring out 2 mg of the sample, adding 1 mL of 2 mol/L trifluoroacetic acid solution, sealing the tube, performing hydrolysis at 105 °C for 10 h, vacuum drying the sample, and finally, derivatizing the sugar sample post-hydrolysis. The standard solution comprising eight varying concentrations of mixed monosaccharides (D-Man, L-Rha, D-Glca, D-Glc, D-Gal, D-Xyl, L-Ara, and L-Fuc) was derivatized to establish a standard curve. The mobile phase consisted of distilled water containing 0.60% potassium dihydrogen phosphate–0.50% triethylamine buffer (phase A) and 99% acetonitrile (phase B). An Agilent C18 column (250 × 4.6 mm, 5 μm) was utilized along with a Phenomeex C18 guard column (10 × 4.6 mm, 5 μm; Phenomeex in Torrance, CA, USA) for protection. The detection conditions for high-performance liquid chromatography included a sample volume of 30 μL, a column temperature of 30 °C, a flow rate of 0.8 mL/min, and a detection wavelength of 250 nm.

### 2.9. Ultraviolet and Infrared Spectroscopy Determination

A 1 mg/mL selenium polysaccharide solution was prepared and scanned in the 200–400 nm wavelength range using an A580 AOELAB UV spectrophotometer (AOE Instruments, Shanghai Co., Ltd. (Shanghai, China)) [6]. The potassium bromide pellet method was used for infrared spectroscopy analysis of selenium polysaccharide samples. A total of 1 mg of dried sample was mixed with 100 mg of potassium bromide, ground evenly, and formed into a transparent pellet. A NEXUS670 Fourier transform infrared spectrometer (Thermo Nicolet, Waltham, MA, USA) was used to scan the spectrum in the 500–4000 cm^−1^ range [10].

### 2.10. Analysis of Antioxidant Activity

The antioxidant activity of selenium polysaccharide extracts was assessed by gauging their capacity to scavenge hydroxyl radicals (^•^OH) [15], DPPH radicals (DPPH) [14], and ABTS radicals (ABTS) [25], and their total reducing power [26]. Vitamin C served as a positive control for the comparative analysis of the in vitro antioxidant activity of SePSs.

### 2.11. Statistical Analysis

The experiment was conducted a minimum of three times to guarantee the reliability of the results. Data analysis was carried out using Excel (Microsoft Office Home and Student 2019) and SPSS26.0 software, with significant differences evaluated through a one-way analysis of variance (*p* < 0.05). Depending on the outcomes of the homogeneity of variance test, either the LSD or Dunnett’s T3 method was selected for variance analysis. Experimental data were displayed as mean ± standard deviation. Graphs were generated using Origin 2022 software.

## 3. Results and Discussion

### 3.1. Effect of Extraction Methods on the Recovery of Selenium-Enriched Polysaccharides

#### 3.1.1. Hot Water Extraction

**Figure 1:**
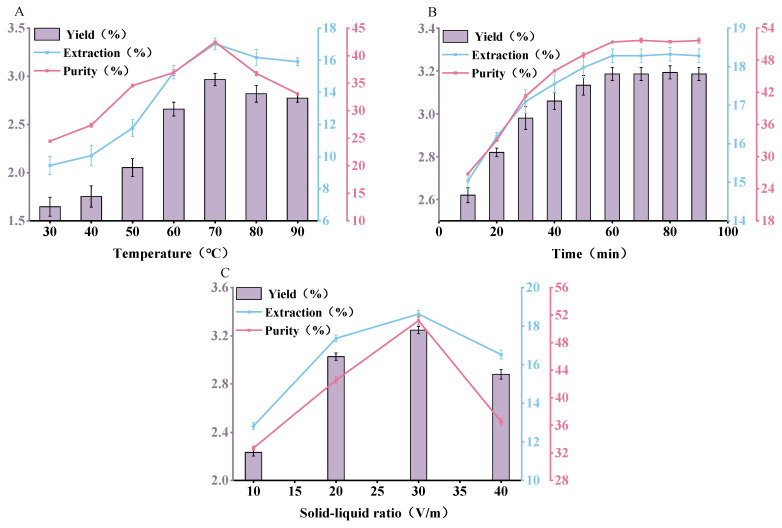
The effect of different extraction conditions during the hot water extraction process on the yield, extraction rate, and purity of SePSs: extraction temperature (**A**), extraction time (**B**), and extraction solid–liquid ratio (**C**) (*p* < 0.05). Processing conditions: (**A**) 20:1 liquid-to-material ratio, extraction for 30 min; (**B**) 70 °C, 20:1 liquid-to-material ratio; and (**C**) 70 °C, extraction for 30 min.

Figure 1A illustrated the impact of extraction temperatures (30~90 °C) on the yield, extraction rate, and purity of selenium-enriched polysaccharides (SePSs). As the temperature progressively increased, the extraction efficiency correspondingly rose. It was suggested that a rise in temperature could expedite the collision frequency among molecules, thereby boosting the diffusion capacity of the molecules and facilitating the dissolution of SePSs into the water to the greatest extent possible. However, once the temperature surpassed 70 °C, the extraction rate of SePSs abruptly declined [27,28,29]. The degradation of SePSs and the subsequent decrease in yield could be attributed to the breaking of chemical bonds in its molecules, which is induced by an increase in temperature, thereby disrupting the structure of the polysaccharide molecules [29,30,31]. Consequently, the optimal water extraction temperature for this experiment was determined to be 70 °C.

Figure 1B shows the effect of extraction time (10~80 min) on the yield, extraction rate, and purity of SePSs, indicating time dependence. The results suggested that the overly long heating may expedite the dissolution of polysaccharides [27,31]. At an extraction time of 60 min, the yield, extraction rate, and purity of SePSs attained a peak state, signifying that the osmotic pressure between the polysaccharides and the water system has achieved equilibrium. Hence, the optimal water extraction time for SePSs was selected to be 60 min.

Figure 1C indicates the impact of liquid-to-material ratios (10:1, 20:1, 30:1, and 40:1, *v*/*w*) on the recovery of SePSs. With all other factors held constant, the yield, extraction rate, and purity of SePSs exhibit a trend of initially increasing and subsequently decreasing as the liquid-to-material ratio escalates. As the liquid-to-material ratio rises from 10:1 to 30:1, it could be due to the fact that a higher liquid-to-material ratio results in a lower concentration and viscosity of the solution during the extraction process, thereby facilitating the dissolution of more polysaccharide molecules into the solvent [27,30]. However, once the liquid-to-material ratio surpassed 30:1, the yield, extraction rate, and purity of SePSs decreased, potentially due to the fact that a larger proportion of solvent could lead to the loss of SePSs [29]. In practical operation, an excess of solvent can complicate the concentration process and extend the concentration time, thereby consuming more time and energy. Consequently, the optimal liquid-to-material ratio in this single-factor hot water extraction experiment is determined to be 30:1.

To summarize, through the single-factor experiment of hot water extraction, the optimal extraction conditions were determined as an extraction temperature of 70 °C, an extraction time of 60 min, and a liquid-to-material ratio of 30:1 (*v*/*w*). Under the optimal extraction conditions (70 °C, 60 min, and 30:1 (*v*/*w*)), the highest yield (3.71 ± 0.02%), extraction rate (21.30 ± 0.09%), and purity (55.16 ± 0.29%) of SePSs were obtained.

#### 3.1.2. Ultrasonic-Assisted Extraction

**Figure 2:**
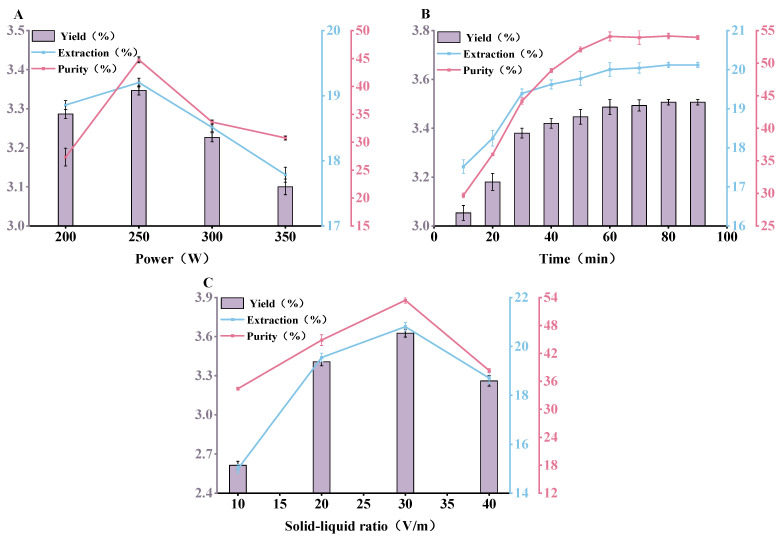
The effect of different extraction conditions in the ultrasound-assisted extraction process on the yield, extraction rate, and purity of SePSs: extraction power (**A**), extraction time (**B**), and extraction solid-liquid ratio (**C**) (*p* < 0.05). Processing conditions: (**A**) 20:1 liquid-to-material ratio, extraction for 30 min; (**B**) 250 W, 20:1 liquid-to-material ratio; and (**C**) 250 W, extraction for 30 min.

In the ultrasonic-assisted extraction process, Figure 2A illustrates the impact of ultrasonic powers (200, 250, 300, and 350 W) on the yield, extraction rate, and purity of SePSs. As the ultrasonic power progressively improved, the extraction efficiency correspondingly rose, but beyond 250 W, the yield, extraction rate, and purity of SePSs gradually declined. This could be attributed to the fact that as the power escalated, the high-intensity shear force of the ultrasonic wave could induce the degradation of SePSs, and excessively high ultrasonic power could also disrupt the structure of SePSs and compromise its stability, all potentially leading to a reduction in the extraction rate, yield, and purity of SePSs [23,32]. Consequently, considering the energy consumption and cost, an ultrasonic power of 250 W was chosen.

Figure 2B presents the impact of extraction time (10~80 min) on the recovery of SePSs. As the ultrasonic time lengthened, the yield, extraction rate, and purity of SePSs increased, and beyond 60 min, the increments in yield, extraction rate, and purity essentially stabilized. It was suggested that the leaching process of SePSs is intimately tied to time. With an increase in duration, more polysaccharides are leached out by ultrasonic waves, and the content of SePSs in the solution is correspondingly enhanced. Once the ultrasonic time hits 60 min, the yield of SePSs appears to stabilize. This could be attributed to the fact that the osmotic pressure inside and outside the cells diminishes, the driving force of mass transfer weakens, and the leaching rate of SePSs nears stability [29,33,34]. Hence, an ultrasonic time of 60 min was determined to be the most suitable choice.

Figure 2C indicates the impact of liquid-to-material ratios (10:1, 20:1, 30:1, and 40:1, *v*/*w*) on the yield, extraction rate, and purity of SePSs. As the liquid-to-material ratio elevated from 10:1 to 30:1, the yield and extraction rate of SePS both exhibited an upward trend; however, once the liquid-to-material ratio surpassed 30:1, the yield, extraction rate, and purity of SePSs started to decline. This could be because a larger volume of the extraction liquid allows for more comprehensive contact between the solvent and the leaching material, enabling more water-soluble polysaccharides to dissolve within the same timeframe, thereby enhancing the extraction rate. However, if the liquid-to-material ratio becomes excessively high, the amount of SePSs leached attains saturation, and concurrently, the ability of ultrasonic waves to break cells decreases, leading to a reduction in the degree of cell breakage, and consequently, a decrease in the extraction rate of effective components [29,35,36]. Therefore, a liquid-to-material ratio of 30:1 was selected.

To summarize, through the single-factor experiment of ultrasonic-assisted extraction, the optimal extraction conditions were determined as an extraction ultrasonic power of 250 W, an extraction time of 60 min, and a liquid-to-material ratio of 30:1 (*v*/*w*). Under the optimal extraction conditions, the best yield (3.97 ± 0.07%), extraction rate (22.76 ± 0.40%), and purity (65.56 ± 0.35%) of SePSs were achieved.

### 3.2. Effect of Extraction Methods on the Total and Organic Selenium Content of Polysaccharides

**Figure 3:**
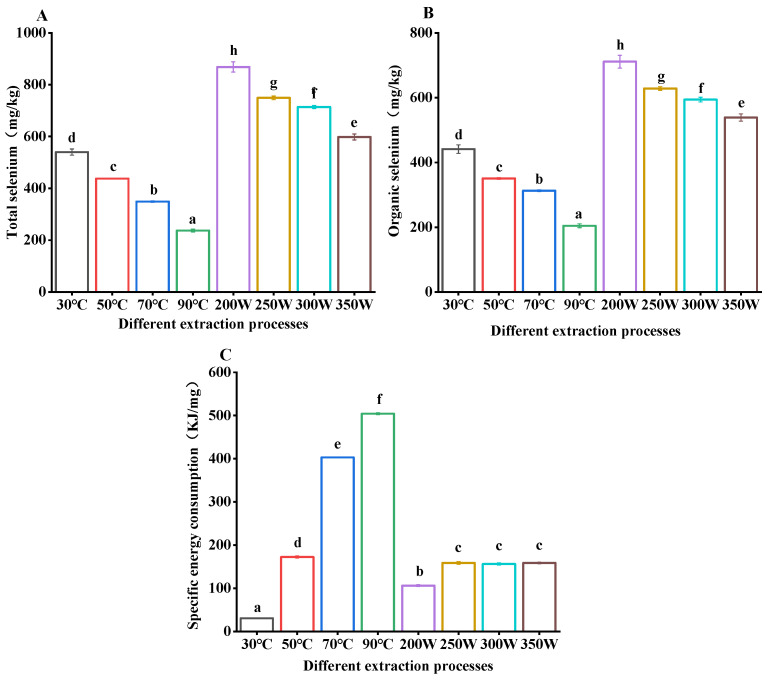
The effect of different extraction processes on the total selenium (**A**), organic selenium (**B**), and specific energy consumption of SePSs (**C**). The meaning of a–h: significance.

The total selenium and organic selenium contents in SePSs extracted from different methods were further analyzed and compared, as depicted in Figure 3A–C. As illustrated in the figure, with a rise in temperature, the specific energy consumption exhibits a gradual upward trend. This could be attributed to the fact that at elevated temperatures, the dissolution, diffusion, and transfer processes become more active, leading to an increase in specific energy consumption [6]. However, beyond a power of 250 W, the specific energy consumption essentially stabilizes. This could be due to the fact that within a certain power range, an increase in extraction efficiency might counterbalance the rise in energy consumption, resulting in no notable escalation in specific energy consumption [37].

As the temperature escalates, the total selenium and organic selenium content in SePS exhibit a downward trend. This could be attributed to the fact that at elevated temperatures, selenium might undergo structural changes or there might be a destruction of selenium–oxygen bonds from SePSs, resulting in selenium loss [6,38]. The presence of selenium in polysaccharides is typically via hydrogen bonds or exists within polysaccharides in the form of selenoesters. During the extraction process, if the temperature becomes excessively high, it might break these chemical bonds and cause selenium to detach. However, a rise in power might expedite the dissolution, diffusion, and transfer processes, thereby enhancing the extraction efficiency. Thus, if this enhanced efficiency is adequate to counterbalance the potential loss of selenium due to the temperature escalation, the total selenium and organic selenium content might not decline notably [27,38,39].

The yield (2.75 ± 0.04%), specific energy consumption (172.53 ± 2.22 KJ/mg), total selenium (437.82 ± 1.56 mg/Kg), and organic selenium (350.43 ± 2.59 mg/Kg) are achieved at 50 °C; the yield (3.71 ± 0.02%), specific energy consumption (402.91 ± 1.13 KJ/mg), total selenium (348.96 ± 2.64 mg/Kg), and organic selenium (312.74 ± 2.65 mg/Kg) are achieved at 70 °C. At 50 °C, the specific energy consumption is low, while the total selenium and organic selenium content is high; however, the yield is higher at 70 °C. The goal is to obtain a higher selenium content and a higher yield under conditions of low energy consumption. At 50 °C, the ratio of organic selenium to specific energy consumption (350.43/172.53) is 2.03, the ratio of total selenium to specific energy consumption (437.82/172.53) is 2.54, the ratio of yield to specific energy consumption (2.75/172.53) is 0.02, and the ratio of yield to organic selenium (2.75/350.43) is 0.01; at 70 °C, the ratio of organic selenium to specific energy consumption (312.74/402.91) is 0.78, the ratio of total selenium to specific energy consumption (348.96/402.91) is 0.87, the ratio of yield to specific energy consumption (3.71/402.91) is 0.01, and the ratio of yield to organic selenium (3.71/312.74) is 0.01. Since the yield, specific energy consumption, and organic selenium ratio at 50 °C are higher than at 70 °C, it is determined that 50 °C is the most suitable extraction temperature during the hot water extraction process. During the ultrasonic-assisted extraction process, the yield, extraction rate, and purity at an extraction power of 250 W are the highest, and the changes in specific energy consumption, total selenium, and organic selenium content are not too pronounced. Consequently, it was further proved that 250 W is the most suitable extraction power during the ultrasonic-assisted extraction process.

Optimal conditions for both hot water and ultrasonic-assisted extraction methods were determined via single-factor experiments, as depicted in Table 1. During hot water extraction, temperatures of both 50 °C and 30 °C were employed. This was done to align with the ultrasonic-assisted extraction method, which utilizes a temperature of 30 °C, thereby facilitating a comparison.

### 3.3. Component Analysis of Different Selenium-Enriched Polysaccharides

A chemical composition analysis was performed, comparing the SePSs extracted from different methods, as presented in Table 2. Ultrasonic treatment has the potential to enhance the levels of neutral sugars, organic selenium, and total selenium in SePSs. It is possible that the strong mechanical and thermal effects produced by ultrasonic waves have the capacity to disrupt the material structure and liberate additional neutral sugars and organic selenium [6]. Moreover, the ultrasound might lead to the degradation of aldonic acid in selenium-enriched *Cardamine violifolia*. It was found that a rise in temperature augmented the presence of aldonic acid in SePSs. This could be attributed to the fact that an elevated temperature enhances the thermal energy of the molecules, enabling a greater number of molecules to surpass the activation free energy, thus escalating the likelihood of molecular collisions and accelerating the reaction rate [5]. Proteins in SePSs might be present as glycoproteins, which were linked by sugars and proteins, and ultrasonic treatment could induce the hydrolysis of glycosidic bonds, consequently influencing the protein content in the extract. This primarily results from the potent thermal and mechanical effects generated by ultrasonic waves. These effects have the potential to disrupt the material structure, including the glycosidic bonds in glycoproteins. A glycosidic bond is a type of covalent bond that links a carbohydrate molecule to another molecule, which could be a protein. When this glycosidic bond is hydrolyzed, the carbohydrate and protein molecules separate, potentially altering the protein concentration [40].

### 3.4. Particle Size Distribution Analysis

**Figure 4:**
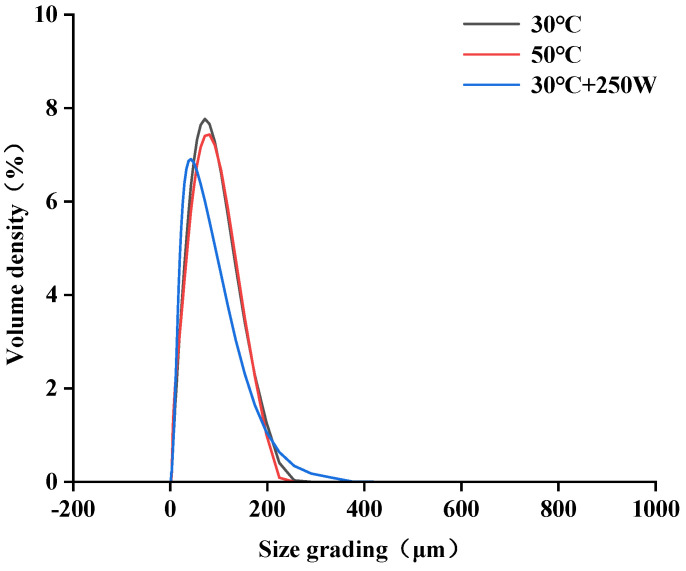
Particle size distribution of SePSs obtained by different extraction methods.

The particle size distribution of SePSs was depicted in Figure 4 and Table 3. The average volume diameter D[4,3] of SePSs, extracted via different methods, exhibited significant variation, with values at 30 °C (63.10 μm), 50 °C (60.93 μm), and 30 °C + 250 W (49.10 μm), respectively. This could be associated with the damage inflicted on SePSs by ultrasonic treatment. Furthermore, the trends of D[3,2], D × 10, D × 50, and D × 90 align with the average volume diameter D[4,3]. It was speculated that ultrasonic treatment might enhance the fragmentation of the extract particles, resulting in a reduction in the dispersibility of polysaccharides [6,41]. When contrasted with the conventional hot water extraction method, ultrasonic assistance has the possibility of significantly reducing the average particle size of polysaccharides. This may come from the cavitation effect of ultrasonic waves, which accelerates the rupture of cell walls, thereby reducing the particle size of polysaccharides [41,42].

### 3.5. Molecular Weight Analysis

**Figure 5:**
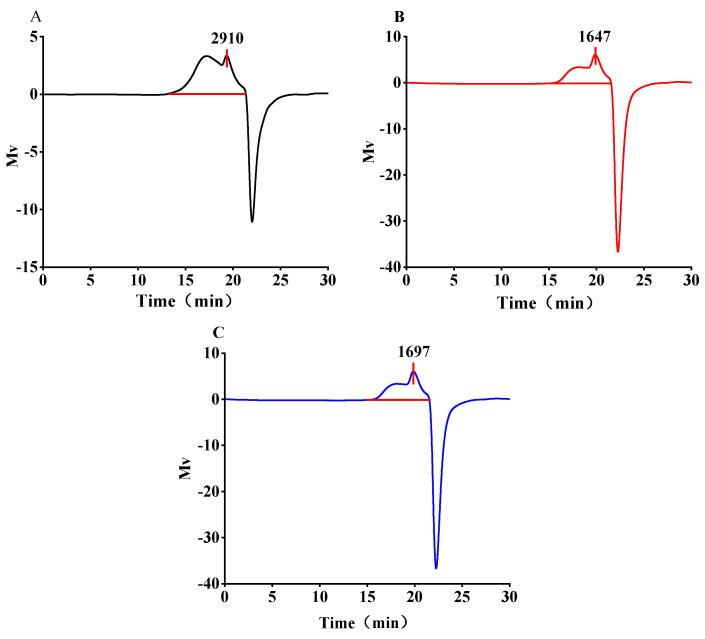
HPGPC chromatogram of SePSs obtained by different extraction methods. GPC chromatograms of different. selenopolysaccharides extracted fromselenium-enriched C. corylifolia leavesat 30 °C (**A**), 50 °C (**B**) and 30 °C + 250 W (**C**).

Gel permeation chromatography (GPC) is employed for the examination of relative molecular weight, where the molecular weight of polysaccharides is inversely proportional to the retention time; a smaller retention time corresponds to a larger molecular weight. As indicated in Table 4 and Figure 5, the weight-average molecular weights (Mw) at 30 °C, 50 °C, and 30 °C + 250 W were 7.291 × 10^4^, 2.572 × 10^4^, and 2.397 × 10^4^, respectively, while the Mw/Mn values were 25.055, 14.093, and 11.747. The data revealed a gradual decrease in the dispersion coefficient, suggesting a reduction in the dispersion coefficient of SePSs extracted via ultrasound. Ultrasonic treatment might disrupt the sugar chains in SePSs, resulting in a narrower molecular weight distribution and diminished dispersibility [43]. The HPGPC chromatogram exhibits inverted peaks. This phenomenon could be attributed to the sample’s refractive index being lower than that of the solvent or mobile phase. This might happen if air is introduced during the sampling process, or if the sample components’ absorption is lower than the mobile phase. Alternatively, the issue could lie in the parameter settings, like errors in the detector signal output settings.

### 3.6. Monosaccharide Composition Analysis

**Figure 6:**
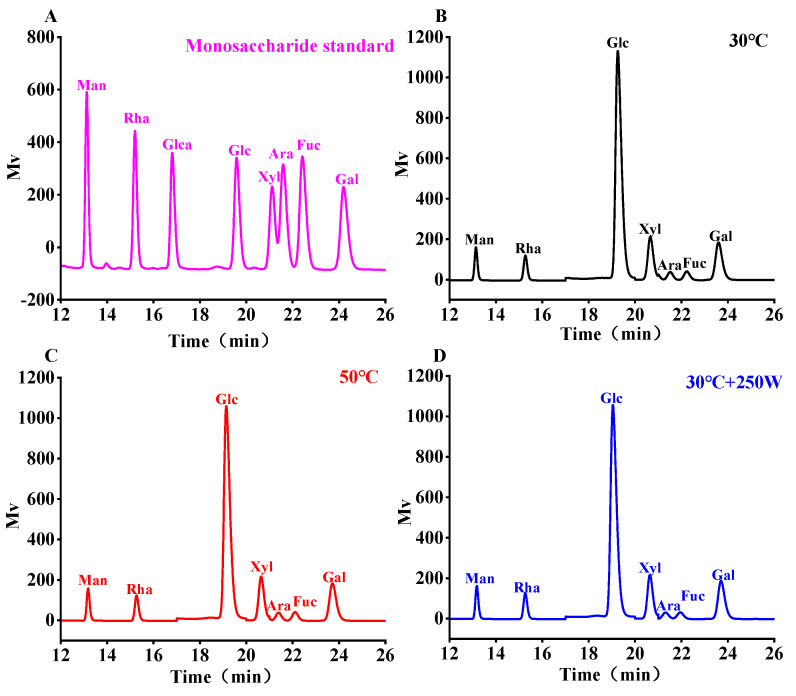
Chromatogram of SePSs obtained by different extraction methods. Monosaccharide composition of different selenopolysaccharides extracted from Cardamine violifolia leaves at various conditions (monosaccharide standard curve (**A**), 30 °C (**B**), 50 °C (**C**), and 30 °C + 250 W (**D**)).

The monosaccharide composition of SePSs, extracted via hot water extraction and ultrasonic-assisted extraction, were depicted in Figure 6 and Table 5. A comparison of eight monosaccharide standards in the liquid chromatogram revealed that all samples contained mannose, rhamnose, glucose, xylose, arabinose, fucose, and galactose, but there was an absence of glucuronic acid. The specific contents of various monosaccharides were calculated based on the peak area from the liquid chromatogram. The results indicated that ultrasonic treatment did not alter the types of monosaccharides in selenium-enriched *Cardamine violifolia*, while it exerted a minor influence on the percentage. It was proposed that ultrasonic treatment primarily impacts the molecular chain of polysaccharides rather than the structure of monosaccharides, leading to the breakage of the polysaccharide molecular chain and an increase in the number of monosaccharides. However, due to constraints related to processing intensity and duration, its effect on the content of monosaccharides is relatively limited [44].

### 3.7. Ultraviolet and Infrared Spectroscopy Analysis

**Figure 7:**
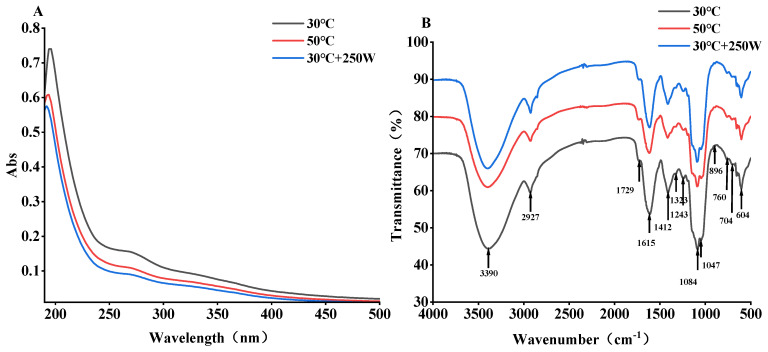
UV (**A**) and FT−IR (**B**) spectra of SePSs obtained by different extraction methods.

In UV scanning, the absorption peaks for nucleic acids and proteins are observed at 260 nm and 280 nm, respectively [45]. It was revealed in Figure 7A that the characteristic absorption peaks at 260 nm and 280 nm were not prominent, suggesting the presence of negligible or minimal amounts of nucleic acids and proteins [46]. The spectral absorption peaks at 260 nm and 280 nm at 30 °C and 50 °C exceed those at 250 W, implying a higher protein content at 30 °C and 50 °C compared to 250 W. As protein concentration is directly proportional to its absorbance, an increase in absorbance signifies a higher protein concentration. Consequently, the relative protein content in different extracts is ordered as follows: 30 °C > 50 °C > 250 W. This order aligns with the composition results presented in Table 2.

As depicted in Figure 7B, the SePSs extracted from three methods exhibited nearly identical characteristic absorption peaks. The pronounced absorption peak at 3390 cm^−1^ signifies the stretching vibration of the hydroxyl group, indicative of O-H groups; the absorption peak at 2927 cm^−1^ corresponds to the stretching vibration of methyl or methylene C-H. Concurrently, a prominent absorption peak was observed at approximately 1729 cm^−1^, and the absorption peak at 1615 cm^−1^ was also intensified. This could be attributed to the formation of Se=O and its subsequent interaction with C=O [33,47]. The less pronounced peaks observed at 1412 cm^−1^ and 1323 cm^−1^ also align with the stretching vibration of C-H in the polysaccharide structure. The peaks discerned at 1243 cm^−1^, 1084 cm^−1^, and 1047 cm^−1^ could be ascribed to the symmetric stretching vibration of the Se–O–C bond [27,47]. The absorption peak at 896 cm^−1^ suggests that the molecular structure of SePSs extracted at 30 °C, 50 °C, and 30 °C + 250 W predominantly comprises β-configuration glycosidic bonds [48]. The region near 760 cm^−1^ is associated with the four adjacent hydrogen atoms on the benzene ring, indicative of the presence of aromatic amino acids. The absorption peak around 604 cm^−1^ suggests the presence of phenolic compounds in SePSs [49]. It was implied that heating and ultrasonic extraction exert no substantial impact on the preliminary structure of polysaccharides.

### 3.8. In Vitro Antioxidant Activity Analysis

**Figure 8:**
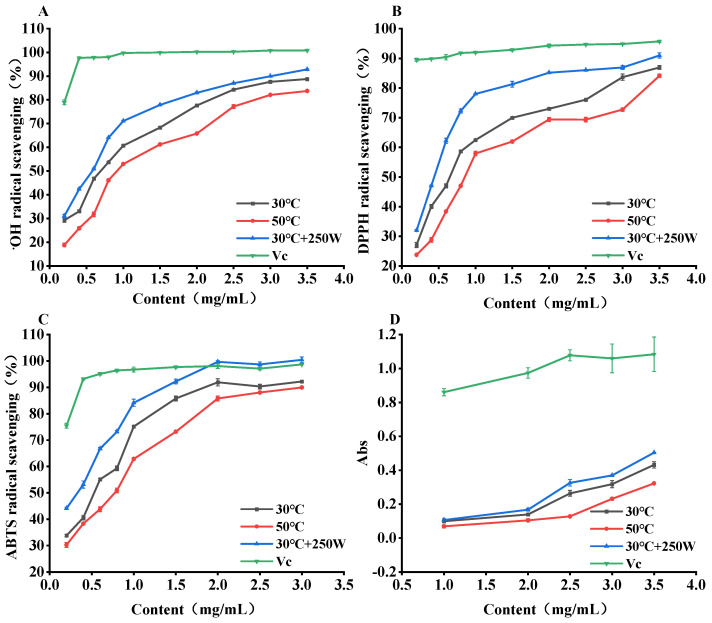
Hydroxyl radical scavenging rate (**A**), DPPH radical scavenging rate (**B**), ABTS radical scavenging rate (**C**), and total reducing power (**D**) of SePSs obtained by different extraction methods.

The antioxidant activity of SePSs in this study follows the order of 30 °C + 250 W > 30 °C > 50 °C. The antioxidant activities of SePSs extracted from different methods are depicted in Figure 8, compared with vitamin C (Vc). From 0.2 to 3.5 mg/mL, the clearance rates for SePSs with ^•^OH, DPPH, and ABTS^•^ escalated with increasing concentration, exhibiting a concentration-dependent pattern. Moreover, the clearance effect of polysaccharides on the ^•^OH and DPPH radicals were less pronounced compared to Vc. However, SePSs extracted under the conditions of 30 °C + 250 W demonstrated a superior ability to scavenge ABTS^•^ than Vc. A lower IC_50_ value corresponds to a higher clearance rate and a more potent clearance ability. As per Table 6, the IC_50_ values for the ^•^OH, DPPH, and ABTS^•^ clearance of SePS extracted under the conditions of 30 °C + 250 W were 0.485 mg/mL, 0.386 mg/mL, and 0.316 mg/mL, respectively. These values were all lower than those of SePSs extracted at 30 °C and 50 °C, while the SePSs extracted at 30 °C exhibited lower values than those at 50 °C. As depicted in Figure 8D, with an increasing concentration of SePSs and Vc, the total reduction power progressively improved. At a concentration of 6.0 mg/mL, the peak absorbance values for 30 °C, 50 °C, 30 °C + 250 W, and Vc reached 0.84, 0.80, 1.01, and 1.22, respectively. Among the three conditions, none exhibits a total reducing power as potent as that of Vc. However, the condition at 30 °C + 250 W demonstrates the highest total reducing power, which approaches that of Vc. It was indicated that SePSs exhibited dual activities of selenium and polysaccharides, since selenium could serve as an antioxidant that efficiently eliminates free radicals and detrimental substances in the body [7,50]. Furthermore, these compounds suggest enhanced antioxidant, anti-tumor, immune regulation, blood sugar reduction, and heavy metal clearance capabilities in comparison to inorganic selenium, polysaccharides, or a blend of inorganic selenium and polysaccharides [50,51]. This could be due to the fact that the amalgamation of selenium and polysaccharides yields more pronounced biological effects, which are advantageous for the body’s absorption and utilization, and exhibit minimal side effects. Consequently, an elevated selenium content in SePSs will correspond to a more potent capacity to eliminate free radicals and detrimental substances [25,26]. The SePSs, extracted under conditions of 30°C and 250 W, demonstrate a significant antioxidant capacity. This capacity may be attributed to their protective effect induced by selenium modification.

## 4. Conclusions

This investigation explored the selenium-enriched residue of *Cardamine violifolia*, post water protein extraction, as the raw material and extracted SePSs utilizing hot water extraction and ultrasonic-assisted extraction techniques. Via single-factor experiments, the optimal extraction process conditions for the two extraction methods were identified, and comparative analyses were performed using the yield, extraction rate, and purity of SePSs as benchmarks. Additional comparative analyses were carried out to evaluate the impacts of hot water extraction and ultrasonic-assisted extraction on the specific energy consumption, total selenium, and organic selenium content of SePSs. The findings reveal that under both conditions of hot water extraction for 60 min at 50 °C with a liquid-to-material ratio of 30:1, and ultrasonic treatment at 250 W for 60 min with a liquid-to-material ratio of 30:1, the yield, extraction rate, purity, specific energy consumption, organic selenium, and total selenium content of SePSs are 2.75% and 3.97%, 15.79% and 22.76%, 49.10% and 65.56%, 172.53 KJ/mg and 158.67 KJ/mg, 350.43 mg/kg and 628.37 mg/kg, and 438.82 mg/kg and 749.16 mg/kg, respectively. Utilizing indicators such as particle size, molecular weight, monosaccharide composition, infrared spectrum, ultraviolet spectrum, etc., it is demonstrated that ultrasound diminishes the particle size and molecular weight during the extraction process. The antioxidant activity of SePSs was investigated via ^•^OH, DPPH, and ABTS, and its total reduction capacity was assessed. The findings indicate that the SePSs prepared under the conditions of ultrasonic 30 °C + 250 W exhibit enhanced antioxidant activity and total reduction capacity. This could be attributed to the fact that the amalgamation of selenium and polysaccharides yields more pronounced biological effects, which are advantageous for the body’s absorption and utilization, and exhibit minimal side effects. Higher selenium content in SePSs enhances their ability to scavenge free radicals and harmful substances. In vitro studies demonstrate that SePSs perform excellently in various antioxidant assays. For instance, SePSs significantly scavenge DPPH, ABTS, and hydroxyl radicals, while enhancing reducing power. These results indicate that SePSs differ from regular polysaccharides in chemical structure and exhibit significantly enhanced antioxidant activity. SePSs are complexes of selenium and polysaccharides and are an important form of organic selenium, where selenium and polysaccharides are connected by covalent bonds. SePSs exist in various forms, including natural, chemically synthesized, microbially transformed, and plant-transformed SePSs, possessing antioxidant, antitumor, immune-enhancing, blood sugar-regulating, and heavy metal-excreting functions. As a kind of functional food additive, SePSs have broad application prospects in food, medicine, and health products. Current research on the biological activity of SePSs primarily uses in vitro experiments. Future studies should employ cell and in vivo experiments to further explore their biological activity. In conclusion, this investigation establishes the groundwork for the application of SePSs in functional foods and offers valuable references for the continued research and development of SePSs.

## Figures and Tables

**Table 1:** Comparison of the effects of hot water extraction and ultrasound-assisted extraction on the yield, extraction rate, purity, specific energy consumption, organic selenium, and total selenium of SePSs.

Extraction Methods	Hot Water Extraction		Ultrasonic-Assisted Extraction
Temperature (°C)	30	50	30
Power (W)	/	/	250
Time (min)	60	60	60
Solid–liquid ratio (g/mL)	30:1	30:1	30:1
Yield (%)	2.45 ± 0.04 ^a^	2.75 ± 0.04 ^b^	3.77 ± 0.04 ^c^
Extraction rate (%)	14.05 ± 0.25 ^a^	15.79 ± 0.20 ^b^	21.61 ± 0.22 ^c^
Purity (%)	46.99 ± 0.16 ^a^	49.10 ± 0.12 ^b^	65.56 ± 0.50 ^c^
Specific energy consumption (KJ/mg)	30.70 ± 0.55 ^a^	172.53 ± 2.23 ^b^	150.67 ± 1.51 ^c^
Organic selenium (mg/Kg)	441.30 ± 13.12 ^a^	350.43 ± 2.59 ^b^	628.37 ± 5.83 ^c^
Total selenium (mg/Kg)	539.10 ± 11.75 ^a^	437.82 ± 1.56 ^b^	749.16 ± 6.91 ^c^

Note: Different lowercase letters in the same row indicate significant differences in their corresponding values (*p* < 0.05).

**Table 2:** Chemical composition of SePSs obtained by different extraction methods.

Sample	Neutral Sugar (%)	Neutral Sugar (%)	Reducing Sugar (%)	Protein Duo (%)	Polyphenol (%)	Organic Selenium (mg/Kg)	Total Selenium (mg/kg)
30 °C	46.99 ± 0.12 ^a^	11.66 ± 0.78 ^a^	0.55 ± 0.02 ^a^	0.59 ± 0.01 ^a^	2.79 ± 0.21 ^a^	441.30 ± 13.12 ^a^	539.10 ± 11.75 ^a^
50 °C	49.10 ± 0.12 ^b^	13.06 ± 0.43 ^b^	0.52 ± 0.02 ^a^	0.46 ± 0.02 ^b^	1.94 ± 0.16 ^b^	350.43 ± 2.59 ^b^	437.82 ± 1.56 ^b^
30 °C + 250 W	65.56 ± 0.35 ^c^	10.73 ± 0.66 ^a^	0.51 ± 0.03 ^a^	0.35 ± 0.02 ^c^	2.39 ± 0.14 ^a^	628.37 ± 5.83 ^c^	749.16 ± 6.91 ^c^

Note: Different lowercase letters in the same row indicate significant differences in their corresponding values (*p* < 0.05).

**Table 3:** Particle size distribution of SePSs obtained by different extraction methods.

Sample	D[4,3] (μm)	D[3,2] (μm)	D × 10 (μm)	D × 50 (μm)	D × 90 (μm)
30 °C	63.10 + 0.29 ^a^	34.83 + 0.12 ^a^	13.73 + 0.12 ^a^	53.73 + 0.09 ^a^	126.67 + 0.94 ^a^
50 °C	60.93 + 0.09 ^b^	31.53 + 0.31 ^b^	12.20 + 1.38 ^ab^	51.67 + 0.25 ^b^	125.33 + 0.47 ^a^
30 °C + 250 W	49.10 + 1.30 ^c^	26.00 + 0.43 ^c^	11.03 + 0.17 ^b^	39.43 + 0.74 ^c^	103.00 + 1.24 ^b^

Note: Different lowercase letters in the same row indicate significant differences in their corresponding values (*p* < 0.05).

**Table 4:** Molecular weight distribution of SePSs obtained by different extraction methods.

Sample	Retention Time (min)	Mn (Da)	Mw (Da)	Mp (Da)	Mw/Mn	Area	Area (%)
30 °C	19.195	2.910 × 10^3^	7.291 × 10^4^	2.095 × 10^3^	25.055	1.300 × 10^6^	100.00
50 °C	19.867	1.825 × 10^3^	2.572 × 10^4^	1.647 × 10^3^	14.093	1.067 × 10^6^	100.00
30 °C + 250 W	19.842	2.039 × 10^3^	2.397 × 10^4^	1.697 × 10^3^	11.747	1.347 × 10^6^	100.00

**Table 5:** Monosaccharide types and percentages in SePSs obtained by different extraction methods.

Sample	Mannose (%)	Rhamnose (%)	Glucose (%)	Xylose (%)	Arabinose (%)	Fucose (%)	Galactose (%)
30 °C	1.94	3.27	22.39	3.40	0.21	1.03	4.99
50 °C	1.92	3.46	22.28	3.33	0.22	1.03	5.02
30 °C + 250 W	1.98	3.55	23.87	3.38	0.21	1.07	5.07

**Table 6:** Measurement results of the half-clearance mass concentration of SePSs obtained by different extraction methods.

Radicals	IC_50_		
	30 °C (mg/mL)	50 °C (mg/mL)	30 °C + 250 W (mg/mL)
^•^OH	0.634 ± 0.004	0.943 ± 0.001	0.485 ± 0.008
DPPH	0.608 ± 0.006	0.873 ± 0.008	0.386 ± 0.002
ABTS	0.454 ± 0.002	0.591 ± 0.003	0.316 ± 0.004

## Data Availability

Data will be made available on request.
